# Dual role of HupF in the biosynthesis of [NiFe] hydrogenase *in Rhizobium leguminosarum*

**DOI:** 10.1186/1471-2180-12-256

**Published:** 2012-11-08

**Authors:** Marta Albareda, Hamid Manyani, Juan Imperial, Belén Brito, Tomás Ruiz-Argüeso, August Böck, Jose-Manuel Palacios

**Affiliations:** 1 Centro de Biotecnología y Genómica de Plantas (C.B.G.P.), Universidad Politécnica de Madrid, Campus de Montegancedo, Carretera M40- km 37.7, 28223 Pozuelo de Alarcón, Madrid, Spain; 2 Departamento de Biotecnología, Escuela Técnica Superior de Ingenieros, Agrónomos, Universidad Politécnica de Madrid, Ciudad Universitaria s/n, 28040 Madrid, Spain; 3 Consejo Superior de Investigaciones Científicas (CSIC), Centro de Biotecnología and Genómica de Plantas (C.B.G.P.), Universidad Politécnica de Madrid (U.P.M.), Madrid, Spain; 4 Department Biology I, University of Munich, Munich, Germany; 5 Current address: ResBioAgro, S.L., Centro de Investigación, Tecnología e Innovación, Universidad de Sevilla, 41012, Sevilla, Spain

**Keywords:** Metalloenzyme, [NiFe] cofactor, Nitrogen fixation, Hydrogenase

## Abstract

**Background:**

[NiFe] hydrogenases are enzymes that catalyze the oxidation of hydrogen into protons and electrons, to use H_2_ as energy source, or the production of hydrogen through proton reduction, as an escape valve for the excess of reduction equivalents in anaerobic metabolism. Biosynthesis of [NiFe] hydrogenases is a complex process that occurs in the cytoplasm, where a number of auxiliary proteins are required to synthesize and insert the metal cofactors into the enzyme structural units. The endosymbiotic bacterium *Rhizobium leguminosarum* requires the products of eighteen genes (*hupSLCDEFGHIJKhypABFCDEX*) to synthesize an active hydrogenase. *hupF* and *hupK* genes are found only in hydrogenase clusters from bacteria expressing hydrogenase in the presence of oxygen.

**Results:**

HupF is a HypC paralogue with a similar predicted structure, except for the C-terminal domain present only in HupF. Deletion of *hupF* results in the inability to process the hydrogenase large subunit HupL, and also in reduced stability of this subunit when cells are exposed to high oxygen tensions. A Δ*hupF* mutant was fully complemented for hydrogenase activity by a C-terminal deletion derivative under symbiotic, ultra low-oxygen tensions, but only partial complementation was observed in free living cells under higher oxygen tensions (1% or 3%). Co-purification experiments using *Strep*Tag-labelled HupF derivatives and mass spectrometry analysis indicate the existence of a major complex involving HupL and HupF, and a less abundant HupF-HupK complex.

**Conclusions:**

The results indicate that HupF has a dual role during hydrogenase biosynthesis: it is required for hydrogenase large subunit processing and it also acts as a chaperone to stabilize HupL when hydrogenase is synthesized in the presence of oxygen.

## Background

[NiFe] hydrogenases are enzymes that catalyze the oxidation of hydrogen into protons and electrons, to use H_2_ as energy source, or the production of hydrogen through proton reduction, as an escape valve for the excess of reduction equivalents in anaerobic metabolism. These enzymes, described in a wide variety of microorganisms, contain two subunits of *ca.* 65 and 30 kDa, respectively. The hydrogenase large subunit contains the active center of the enzyme, a heterobimetallic [NiFe] cofactor unique in nature, in which the Fe atom is coordinated with two cyano and one carbonyl ligands; the hydrogenase small subunit contains three Fe-S clusters through which electrons are conducted either from H_2_ to their primary acceptor (H_2_ uptake), or to protons from their primary donor (H_2_ evolution) [1].

Biosynthesis of [NiFe] hydrogenases is a complex process that occurs in the cytoplasm, where a number of auxiliary proteins are required to synthesize and insert the metal cofactors into the enzyme structural units [2]. In most *Proteobacteria,* genetic determinants for hydrogenase synthesis are arranged in large clusters encoding *ca*. 15–18 proteins involved in the process. Most hydrogenase genes are conserved in different proteobacterial hydrogenase systems, suggesting an essentially conserved mechanism for the synthesis of these metalloenzymes [3]. The biosynthesis of the hydrogenase [NiFe] cofactor and its transfer into the hydrogenase large subunit have been thoroughly studied in the *Escherichia coli* hydrogenase-3 system [2]. In that system, cyano ligands are synthesized from carbamoylphosphate through the concerted action of HypF and HypE proteins [4,5] and transferred to an iron atom exposed on a complex formed by HypC and HypD proteins [6]. The source and biosynthesis of the CO ligand likely follows a different path [7-9] whose details are still unknown, although recent evidence suggests that gaseous CO and an intracellular metabolite might be sources for the ligand [10]. When the iron is fully coordinated, HypC transfers it to pre-HycE, the precursor of the large subunit of *E. coli* hydrogenase-3. After incorporation of the precursor cofactor into HycE, proteins HypA, HypB, and SlyD mediate Ni incorporation into the active site [11]. After nickel insertion, the final step is the proteolytic processing of the hydrogenase large subunit by a nickel-dependent specific protease [12].

Hydrogen is produced in soils as a result of different metabolic routes. A relevant source of this element is the process of biological nitrogen fixation, in which at least 1 mol of hydrogen is evolved per mol of nitrogen fixed as a result of the intrinsic mechanism of nitrogenase [13]. As a consequence, many diazotrophic bacteria, including some rhizobia, induce [NiFe] hydrogenases along with nitrogenase to recover part of the energy lost as hydrogen [14]. The genome of the legume endosymbiotic bacterium *Rhizobium leguminosarum* bv. viciae UPM791 encodes a single hydrogenase that is expressed under symbiotic conditions by the concerted action of eighteen genetic determinants (*hupSLCDEFGHIJKhyp-ABFCDEX)* clustered on the symbiotic plasmid [15]. Symbiotic expression of hydrogenase structural genes (*hupSL)* is controlled by the NifA-dependent promoter P_1_[16]. In addition, an FnrN-type promoter controls the expression of the *hypBFCDEX* operon under microaerobic and symbiotic conditions [17]. For practical purposes, the NifA-dependent *hupSL* promoter has been replaced by the FnrN-dependent *fixN* promoter (P_*fixN*_), thus allowing expression of hydrogenase in microaerobic vegetative cells [18]. A single FnrN-dependent promoter drives the expression of *hupSL* and all downstream hydrogenase genes in cosmid pALPF1. This plasmid and its deletion derivatives, along with the *hup*-deleted *R. leguminosarum* strain UPM 1155, have been used as a model to study hydrogenase synthesis in this bacterium [19].

The *R. leguminosarum* hydrogenase cluster encodes two proteins (HupF and HupK) not present in *E. coli* but conserved in other hydrogenase systems such as those from *Ralstonia eutropha*[20], *Bradyrhizobium japonicum*[21], and *Rhodobacter capsulatus*[22]. In the case of *Thiocapsa roseopersicina*, HupK and two copies of HypC have been described [23].

HupF is a paralog of HypC but, apart from this, no further data are available on the function of this protein in the *R. leguminosarum* system. HoxL, the HupF homolog in the *R. eutropha* system, is essential for the synthesis of active hydrogenase [20]. Recently, a model has been proposed for the synthesis of the oxygen-tolerant hydrogenase from *R. eutropha*[24]. According to this model, the interaction between HoxV, the HupK homolog in that system, and HypC plays a key role as intermediate able to accommodate the Fe(CN^-^)_2_CO cofactor precursor from the HypCD complex prior to its incorporation into a complex containing the hydrogenase large subunit (HoxG) and HoxL [20]. This model is further supported by the fact that HypC2 from *T. roseopersicina* was able to interact with HupK and HypD [23].

In this work we present evidence indicating that *R. leguminosarum* chaperone HupF has a second role in hydrogenase biosynthesis: in addition to its proposed role in assisting the transfer of Fe-containing precursor cofactor from HupK to HupL, it plays a protective role on hydrogenase structural subunit HupL when cells are exposed to oxygen.

## Results

### The existence of *hupF* and *hupK* correlates with the presence of *hypC* in the genome of aerobic bacteria

A BLAST search for homologues to *R. leguminosarum* HupF and HupK proteins in a set of 408 completed genomes from *Proteobacteria* in the NCBI database revealed the presence of two *hupF*/*hypC*-like genes in 21 out of 77 proteobacterial genomes encoding [NiFe] hydrogenases. In all these cases, a *hupK*-like gene was identified in the DNA region between *hupF* and *hypC* (Table 1) suggesting a structure for hydrogenase gene clusters similar to that described for *R. leguminosarum*[15]. Interestingly, all organisms encoding the three HupF, HypC and HupK proteins were able to express hydrogenase in the presence of oxygen. Anaerobic bacteria (sulphate-reducers and other anaerobes) encoded only one *hypC/hupF*-like gene, and no *hupK*-like gene, and the same situation was found in *Enterobacteriaceae*.

**Table 1:** **Location of genes encoding HupL, HupF, HupK, and HypC proteins in genomes from *Proteobacteria***

**Bacterial species**	**#**^**a**^	**KEGG**^**b**^**Locus designation for homolog to**			
		**HupL**	**HupF**	**HupK**	**HypC**
*Alkalimnicola ehrlichei*	1	Mlg_2028	Mlg_2025	Mlg_2020	Mlg_2016
*Azoarcus* sp. BH72	2	azo3787	azo3793	azo3798	azo3802
*Azotobacter vinelandii*	3	Avin_50580	Avin_50550	Avin_50500	Avin_50460
*Beijerinckia indica*	4	Bind_1151	Bind_1154	Bind_1158	Bind_1162
*Bradyrhizobium sp*. ORS278	5	BRADO1685	BRADO1688	BRADO1693	BRADO1698
*Bradyrhizobium japonicum* USDA110	6	bsl6941	bsl6938	bll6933	bsl6929
*Bradyrhizobium sp*. BTAi1	7	BBta_1997	BBta_2000	BBta_2005	BBta_2009
*Burkholderia vietnamiensis*	8	Bcep1808_5932	Bcep1808_5935	Bcep1808_5940	Bcep1808_5944
*Burkholderia phymatum*	9	Bphy_7264	Bphy_7261	Bphy_7257	Bphy_7253
*Dechloromonas aromatica*	10	Daro_3988	Daro_3985	Daro_3980	Daro_3967
*Magnetococcus* sp.	11	Mmc1_2503	Mmc1_2501	Mmc1_2497	Mmc1_2490
*Magnetospirillum magneticum*	12	amb1647	amb1645	amb1644	amb1640
*Methylibium petroleiphilum*	13	Mpe_A2826	Mpe_A2821	Mpe_A2817	Mpe_A2813
*Paracoccus denitrificans*	14	Pden_3098	Pden_3102	Pden_3106	Pden_3110
*Polaromonas naphtalenivorans*	15	Pnap_1974	Pnap_1970	Pnap_1965	Pnap_1961
*Ralstonia metallidurans*	16	Rmet1297	Rmet1292	Rmet1287	Rmet1283
*Rhodobacter sphaeroides* ATCC17029	17	Rsph17029_2147	Rsph17029_2151	Rsph17029_2155	Rsph17029_2159
*Rhodoferax ferrireducens*	18	Rfer_4091	Rfer_4093	Rfer_4118	Rfer_4098
*Rhodopseudomonas palustris*	19	RPA0963	RPA0967	RPA0972	RPA0976
*Rhodospirillum rubrum* ATCC11170	20	Rru_A1162	Rru_A1165	Rru_A1167	Rru_A0307
*Xanthobacer autotrophicus*	21	Xaut_2174	Xaut_2177	Xaut_2181	Xaut_2185

^a^Taken from KEGG gene database ( http://www.genome.jp/kegg/genes.html).

^b^Ordinal numbers used in phylogenetic tree of Figure 1.

**Figure 1:**
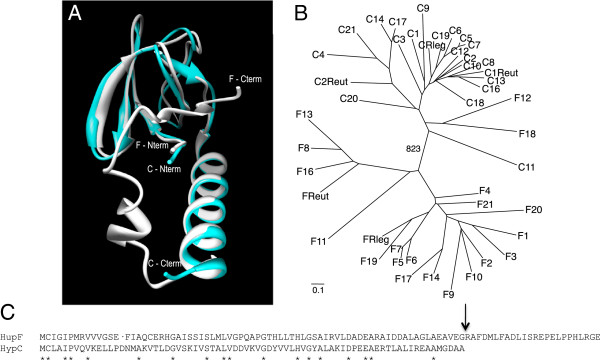
**Structural, phylogenetic, and sequence comparisons of HupF and HypC.A**) Overlay of HupF (white) and HypC (blue) predicted structures. Structural predictions were carried out with the UCSF Chimera package from the Resource for Biocomputing, Visualization, and Informatics at the University of California, San Francisco ( [26]; supported by NIH P41 RR001081), and were based on the structure of *Thermococcus kodakarensis* HypC (PDB 2z1c) and on *ab initio* predictions using the I-TASSER server [27]. Positions of N- and C-termini of each protein are indicated. **B**) Neighbour-joining phylogenetic tree of HupF and HypC. Sequences derived from the *hupF* and *hypC* genes listed in Table 1, along with those from *R. leguminosarum* (FRleg and CRleg) and *R. eutropha* (FReut, C1Reut, and C2Reut), were aligned with ClustalX, and the alignment was corrected for multiple substitutions and refined manually. Distances were generated with the same program using the neighbour-joining method, and bootstrapped (1000x). TREEVIEW was used to draw the most likely tree. Sequence names shown in the tree contain a first letter indicating HupF or HypC protein, followed by a number corresponding to that assigned to each species in Table 1. **C**) Sequence alignment of *R. leguminosarum* HupF and HypC proteins. Alignment was carried out on a structural basis using I-TASSER. Asterisks indicate conserved residues. Vertical arrow indicates the start point for the C-terminal deletion in HupF_CST_.

The availability of the 3D structure of HypC from *Thermococcus kodakarensis*[25] allowed us to model both *R. leguminosarum* HypC and HupF proteins on that template (Figure 1A). We found that the model derived for HupF is compatible with a structure highly similar to that of HypC, except for the C-terminal domain present only in HupF (Figure 1C). This structural similarity suggests a related function for both proteins.

We used the *hupF*/*hypC* sequences identified above to build a phylogenetic tree for this group of proteins (Figure 1B). In this tree we included the sequences corresponding to *hupF* and *hypC* genes shown in Table 1, along with sequences from HupF/HypC-like proteins from the well studied hydrogenase systems from *R. leguminosarum* and *R. eutropha*. Analysis of this phylogenetic tree revealed that HupF clusters as a coherent branch separated from HypC, suggesting a divergent evolution from a common ancestor driven by selection for potential functional differences of the two proteins.

### HupF is required for hydrogenase activity

Previous transposon mutagenesis of the *R. leguminosarum* hydrogenase region did not result in insertions located in *hupF*[28,29]. In order to test the essentiality of this gene for hydrogenase activity we analyzed the hydrogenase activity associated to cosmid pALPF5, a pALPF1 derivative harboring the *hup/hyp* gene cluster with a precise deletion on *hupF* gene (see Methods). In these experiments, microaerobic (1% O_2_) cultures of the *hup*-complete strain UPM 1155(pALPF1) showed high levels of hydrogenase activity, whereas the *hupF*-deleted strain UPM 1155(pALPF5) showed only basal levels of activity similar to those observed for the *hypC*-deleted strain UPM1155(pALPF14) used as negative control (Table 2). The Δ*hupF* mutant was fully complemented by plasmid pPM501, encoding a HupF protein C-terminally fused to a *Strep*TagII affinity tail (HupF_ST_,see Methods section). These data also indicate that HupF_ST_ is fully functional.

**Table 2:** **Hydrogenase activity induced by *R. leguminosarum *strains in microaerobic cultures and in pea bacteroids**

**Strain**	**Genotype**	**Hydrogenase activity**^**a**^		
		**1% O**_**2**_	**3% O**_**2**_	**Bacteroids**
UPM1155(pALPF1)	wild type	13440 ± 1720	4970 ± 472	5980 ± 852
UPM1155 (pALPF14)	Δ*hypC*	< 200	< 200	< 200
UPM1155 (pALPF5)	Δ*hupF*	< 200	< 200	470 ± 84
UPM1155 (pALPF5/pPM501)	Δ*hupF*/*hupF*_ST_	12890 ± 230	5900 ± 779	5350 ± 728
UPM1155(pALPF5/pPM501C)	Δ*hupF*/*hupF*_CST_	4780 ± 228	1025 ± 255	5400 ± 683

^a^Values, expressed in nmoles H_2_ h^-1^ (mg protein)^-1^, are the average of three independent assays ± SE.

### HupF contributes to HupL stability under elevated oxygen tensions

**Figure 2:**
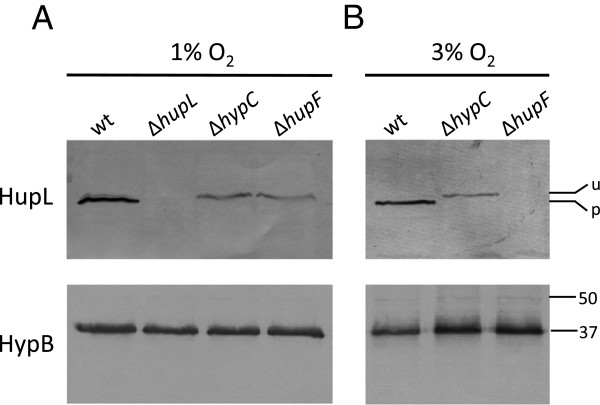
**Effect of oxygen level and presence of HupF on HupL status.** Immunodetection of HupL and HypB proteins was carried out in crude cell extracts from *R. leguminosarum* cultures induced for hydrogenase activity under 1% O_2_ (**A**) or 3% O_2_ (**B**). Strains: UPM1155 derivative strains harboring plasmids pALPF1 (wt), pALPF2 (Δ*hupL*), pALPF14 (Δ*hypC*), and pALPF5 (Δ*hupF*). Proteins were resolved by SDS-PAGE in 9% (top panel) or 12% (bottom panel) acrylamide gels. Each lane was loaded with 60 μg (top panels) or 10 μg (bottom panels) of protein. Marks on the right margin indicate the location of the two forms of HupL protein: unprocessed HupL (u, 66 kDa), processed HupL (p, 65 kDa), or the position of molecular weight markers of the indicated size.

**Figure 3:**
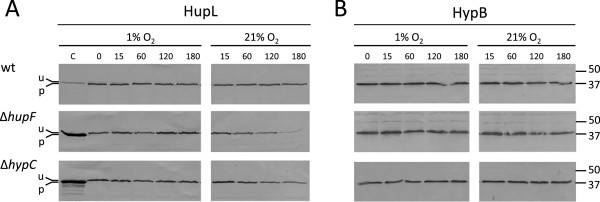
**Effect of HupF on HupL stability under high oxygen tensions.** Time course of immunodetection of HupL (panel **A**) and HypB (panel **B**) proteins in cell crude extracts from cultures previously induced for hydrogenase activity and then bubbled with 1% O2 or air (21% O_2_) for the indicated periods of time (min). Top, medium, and bottom panels correspond to cell extracts from *R. leguminosarum* UPM1155 derivative strains harboring plasmids pALPF1 (wt), pALPF5 (Δ*hupF*), and pALPF14 (Δ*hypC*), respectively. Conditions of SDS-PAGE and loading are as in Figure 2. Lanes labelled as 0 contain control crude extracts harboring either unprocessed HupL from UPM1155(pALPF14) (Δ*hypC*), in top panel, or processed HupL from UPM1155(pALPF14) (wt), in medium and bottom panels as controls. Marks on the left margins indicate the position of the unprocessed (u, 66 kDa) and processed (p, 65 kDa) forms of HupL in panel A, and marks on the right margins indicate the position of molecular weight markers.

The existence of *hupF* in hydrogenase systems from bacteria synthesizing this enzyme in the presence of oxygen prompted us to study the potential role of this protein in protection against oxygen. To this aim, we analyzed the possible effect of HupF on the status of hydrogenase large subunit in cultures maintained under different oxygen tensions (1% and 3%). The higher oxygen tension (3%) still allowed the expression of hydrogenase in *R. leguminosarum* wild-type strain, although at a reduced level (40% of the level induced under 1% O_2_, Table 2). The presence and processing status of the hydrogenase large subunit (HupL) were analyzed in crude cell extracts from microaerobic cultures through immunoblot (Figure 2). In these experiments we found that the wild-type cells contained a clear band associated to the mature form of HupL, irrespective of whether cells were induced under 1% or 3% oxygen (Figure 2A and 2B, upper panel). This band was absent in a Δ*hupL* mutant used as negative control (Figure 2A). Analysis of the cell extracts from the Δ*hupF* strain grown at 1% oxygen revealed the presence of HupL, although in the unprocessed form (Figure 2A, upper panel). Interestingly, HupL was not detected when cultures from the same mutant strain were incubated under 3% O_2_ (Figure 2B). In contrast, extracts from a *R. leguminosarum* mutant lacking HypC, used as a hydrogenase non-processing control, showed a clear band of unprocessed HupL after exposure to both 1% and 3% oxygen tension (Figure 2A and 2B). Similar levels of an immunoreactive band corresponding to HypB were detected in all the extracts (Figure 2, lower panels), indicating that the microaerobic induction of Hup expression was equally effective for all strains in each treatment. These data suggest that, in the presence of 3% oxygen, HupL is either unstable or not synthesized in the absence of HupF. In order to further evaluate these possibilities, we analyzed the *in vivo* stability of HupL as a function of the presence/absence of HupF. To address this question, we first induced *R. leguminosarum* cultures for hydrogenase expression under 1% oxygen, and then the induced cells, carrying either processed HupL (wild-type strain) or unprocessed HupL (Δ*hupF* and Δ*hypC* mutants), were exposed to atmospheres containing either 1% O_2_ or 21% O_2_ for up to 3 hours. After such treatments, the amount and processing status of HupL was determined through immunoblot assay in cell extracts (Figure 3A). The result of these experiments revealed that, as expected, the level of HupL was not significantly altered by incubation under 1% O_2_ in all the strains tested. Also, incubation of wild-type cells under 21% oxygen revealed that the mature form of hydrogenase large subunit was fully stable under these conditions. In contrast, incubation of Δ*hupF* cultures under 21% O_2_ resulted in the gradual disappearance of unprocessed HupL, virtually undetectable after 3 h, whereas the unprocessed form in the Δ*hypC* mutant was significantly more stable upon incubation under 21% oxygen. A similar analysis performed with an anti-HypB antiserum, used as control, revealed that the levels of this protein were stable during the incubation, irrespective of whether cells were incubated under 1% or 21% O_2_ (Figure 3B).

### HupF participates in protein complexes with HupL and HupK during hydrogenase biosynthesis

The observed role of HupF on stabilization of HupL in the presence of oxygen prompted us to examine the existence of interactions between both proteins. We studied such interactions through pull-down experiments with soluble extracts from *R. leguminosarum* cultures expressing HupF_ST_ from plasmid pPM501. In this plasmid the expression of *hupF*_ST_ is under the control of the same P_*fixN*_ promoter used for the remaining *hup/hyp* genes in pALPF1. In order to "freeze" intermediate complexes produced during the biosynthetic process, this plasmid was expressed in strain UPM 1155(pALPF4), carrying an in-frame deletion in the gene (*hupD*) for the protease involved in the final step of HupL maturation.

**Figure 4:**
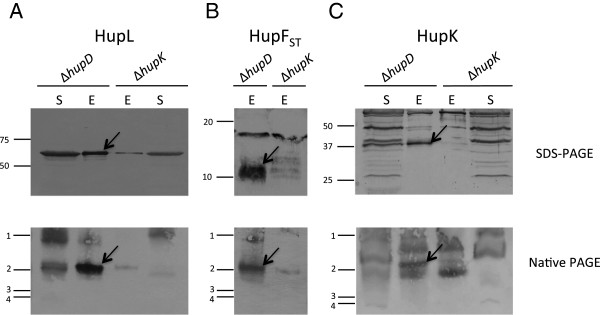
**Pull-down analysis of HupF interactions with HupL and HupK proteins.** Proteins were resolved by SDS-PAGE (top panels) or 4-20% gradient native PAGE (bottom panels). Immunoblots were revealed with antisera raised against HupL (panel **A**) or HupK (panel **C**), or with *Strep*Tactin-alkaline phosphatase conjugate (panel **B**) to detect HupF_ST_. Eluates (E) were obtained from extracts from *R. leguminosarum* UPM 1155 derivative strains harboring pALPF1-derivative plasmids deficient in *hupD* (pALPF4) or in *hupK* (pALPF10) and expressing HupF_ST_ from plasmid pPM501. Soluble extracts (S) of the corresponding cultures were loaded as controls for detection of HupL and HupK proteins. Arrows indicate the relevant bands identified in the eluate from the Δ*hupD* mutant. Proteins subjected to SDS-PAGE (top panels) were loaded in gels with different amounts of polyacrylamide (9% for HupL, 15% for HupF_ST_, and 12% for HupK). Numbers on the left margin of the panels indicate the position of molecular weight standards (kDa, top panels), or the position of BioRad Precision Plus Standards (1, 250 kDa; 2, 150 kDa, 3, 75 kDa; 4, 100 kDa) in native gels (bottom panels).

Soluble fractions from *R. leguminosarum* UPM 1155(pALF4, pPM501) cultures grown under microaerobic conditions (1% O_2_) were loaded into *Strep*Tactin columns, and desthiobiotin-eluted fractions were separated by SDS-PAGE and analyzed through immunoblot (Figure 4, upper panels). When membranes were probed with *Strep*Tactin-AP conjugate, a strong band of the expected size for HupF_ST_ (*ca*. 10 kDa. Figure 4B) was detected, indicating that the system was efficient in recovering this protein. Similar immunoblots were developed with an anti-HupL antiserum. In these experiments we found in the eluates a strong immunoreactive band of a size corresponding to the unprocessed form of the hydrogenase large subunit (*ca.* 66 kDa, Figure 4A). This band could be detected also in the soluble extract. The co-purification of this protein along with HupF_ST_ suggests the existence of a complex between HupF and HupL.

Immunoblot analysis was also carried out with an anti-HupK antiserum (Figure 4C). This analysis identified several immunoreactive bands in the soluble fraction of the Δ*hupD* mutant, one of which likely corresponded to HupK, since it showed the expected molecular size (*ca.* 37 kDa) for this protein*,* and was absent in the extract from the Δ*hupK* mutant. Analysis of the *Strep*Tactin eluates with the same antiserum revealed that the same specific band co-eluted with HupF_ST_ in the Δ*hupD* mutant, but was absent in the eluate from the *hupK*-deficient strain, strongly suggesting the existence of a complex involving HupF and HupK. It has to be noted that eluates obtained from the *hupK*-deficient mutant contained reduced levels of HupF_ST_ (and hence, of coeluted HupL), suggesting that HupF might require the presence of HupK for full stability (Figure 4A and 4B).

In order to obtain additional confirmation for the existence of the complexes deduced from the pull-down experiments described above, the eluates were further analyzed using non-denaturing conditions. To this aim, the immunoblot analysis was repeated after the proteins eluted from *Strep*Tactin columns were resolved in 4-20% gradient polyacrylamide native gels (Figure 4, lower panels). When the immunoblot was developed with anti-HupL antiserum, a major immunoreactive band was detected in eluates from the Δ*hupD* derivative strain (Figure 4A). A band of similar size and mobility was detected when a replicate immunoblot was developed with the *Strep*Tactin-AP conjugate (Figure 4B), suggesting that both bands correspond to a HupL-HupF complex. In both cases, the absence of HupK was associated to the virtual absence of HupF_ST_-containing complexes (Figure 4A and 4B). Finally, a third replicate of the same immunoblot developed with the anti-HupK antiserum revealed a fainter band, with a slightly lower mobility (Figure 4C), suggesting a different, less abundant HupK-HupF complex. As before, non-specific bands were detected by this antiserum in the Δ*hupK* mutant, likely corresponding to complexes of the non-specific bands detected in the SDS-PAGE experiments described above.

Further confirmation on the composition of the complex or complexes detected by immunoblotting was sought by peptide mass fingerprinting analysis of the major complex present in the eluate obtained from the Δ*hupD* strain UPM 1155(pALPF4, pPM501). Such eluate was resolved by 4-20% gradient native PAGE, followed by Coomassie Blue staining. In this gel we identified a clear band with a mobility similar to that of the complexes identified above (data not shown). This band was excised and subjected to MALDI-TOF analysis after trypsin digestion. The analysis led to the identification of peptides corresponding to proteins HupL and HupF (data not shown), indicating the presence of a major complex involving these two proteins. In this analysis no peptides corresponding to HupK, nor to any other Hup/Hyp proteins, were detected. Taken together, data from immunoblot and mass spectrometry analyses suggest the presence of two different complexes: a major complex containing HupF and HupL, and a second, much less abundant complex involving HupF and HupK, only detectable through immunoblot analysis.

### Functional analysis of the HupF C-terminal region

**Figure 5:**
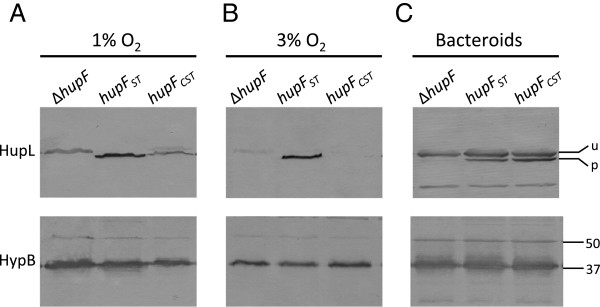
**Effect of a C-terminal deletion on HupF in *R. leguminosarum *hydrogenase processing.** Immunodetection of hydrogenase large subunit HupL (top panels) and HypB (bottom panels) was carried out in crude extracts from vegetative cells induced for hydrogenase activity under different oxygen tensions (1% or 3%), and in bacteroid crude extracts. Strains: *R. leguminosarum* UPM1155 derivatives carrying plasmids pALPF5 (Δ*hupF*), pALPF5/pPM501 (*hupF*_ST_), and pALPF5/pMP501C (*hupF*_*CST*_). Proteins (60 μg for HupL and 10 μg for HypB) were resolved in 9% (HupL) or 12% (HypB) acrylamide SDS-PAGE gels.

A distinctive domain of *R. leguminosarum* HupF is the extended C-terminal region, absent in the otherwise structurally related HypC protein (Figure 1). In order to elucidate the relevance of this region for HupF function, we constructed plasmid pPM501C, a pPM501 derivative in which the *hupF* gene was modified to produce a truncated version of HupF_ST_ (HupF_CST_) with a precise deletion of the C-terminal 24 amino acid residues of HupF (see Methods). When this plasmid was introduced into the HupF-deficient strain UPM 1155(pALPF5), only partial restoration of hydrogenase activity (37%) was observed under standard inducing conditions (1% O_2_, Table 2) and, consistently, the amount of processed protein was significantly reduced (Figure 5A, top panel). These data indicate that the truncated form of the protein is partially impaired in its role when hydrogenase biosynthesis is carried out in an atmosphere of 1% O_2_. Since HupF was shown to contribute to HupL stability under higher oxygen tensions (Figure 2), we also tested the effect of the C-terminal deletion under these conditions. Interestingly, when hydrogenase was induced in an atmosphere containing 3% oxygen, the truncated form of the protein supported only 17% of the activity associated to the complete form of the protein (Table 2), which corresponded to virtually undetectable amounts of processed HupL protein (Figure 5B, top panel). Since the evidence pointed towards a more relevant role for the C-terminal region of HupF under higher oxygen tensions, we hypothesized that such an effect should be less relevant under symbiotic conditions. Bacteroids within the legume nodule are maintained under oxygen tensions in the nanomolar range [30], at least three orders of magnitude lower than those present in microaerobic cultures. We determined hydrogenase activity and HupL processing in pea bacteroids induced by *R. leguminosarum* strains carrying either the whole or the truncated version of HupF. In this experiment, both forms of the protein complemented the Δ*hupF* mutant to wild-type levels of activity, irrespective of the presence of the C-terminal region (Table 2). Also, immunoblot analysis of bacteroid crude extracts indicated that the level of HupL processing was not significantly altered by the deletion (Figure 5C). These data indicate that the C-terminal region of the protein is not required at ultra-low oxygen tensions.

## Discussion

The maturation of metalloenzymes such as [NiFe] hydrogenase requires the biosynthesis and insertion of metal cofactors through the action of auxiliary proteins. The soluble, hydrogen-evolving hydrogenase-3 enzyme from *E. coli* has served as a model to elucidate the intricate biosynthetic pathway for the [NiFe] cofactor [2]. However, there is increasing evidence indicating that the model for biosynthesis of this cofactor might not be universal [20]. Ancient enzymes such as hydrogenase had to evolve to accommodate into an O_2_-containing environment. From a biotechnological point of view, oxygen tolerance is a relevant characteristic with obvious interest [31]. The initial model described for the oxygen-sensitive hydrogenase from *Desulfovibrio gigas*[32] has been enriched by recent crystal structures of oxygen tolerant hydrogenases from *Hydrogenovibrio marinus*, *R. eutropha*, and *E. coli*, showing that in the case of oxygen-tolerant enzymes, the iron-sulfur cluster proximal to NiFe cofactor corresponds to an unprecedented [4Fe3S] type coordinated with six cysteines [33-35]. This cluster provides redox protection to the NiFe cofactor, by allowing the enzyme to catalyze reduction of O_2_ to water “in situ” as well as the oxidation of hydrogen. An oxidative environment may also require protection during enzyme biosynthesis. From a genetic point of view, a relevant variation lies in the presence of two additional genes, *hupF* and *hupK* and their homologues, encoding auxiliary proteins in hydrogenase systems from aerobic bacteria. Using a specific deletion mutant we have shown in this work that HupF is essential for hydrogenase activity in *R. leguminosarum*, as it has been described in the *R. eutropha* system [20]. The results obtained here indicate that HupF has a dual role during hydrogenase biosynthesis: it is required for hydrogenase large subunit processing and also acts as a chaperone to stabilize HupL when hydrogenase is synthesized in the presence of oxygen.

Data from experiments on exposure of HupL-containing cells to different oxygen tensions indicate that, in the absence of HupF, unprocessed HupL gradually disappears at high oxygen tensions. Since there is no P_*fixN*_-driven expression of *hupL* at 21% O_2_[18], the decrease in the level of HupL is likely due to a loss of stability of the protein.

Analysis of the C-terminal deletion mutant of HupF suggests that this domain might be relevant for HupL stabilization and might provide additional support for the role of HupF as an oxygen protective chaperone. The C-terminally truncated protein is functionally indistinguishable from the full-size protein under symbiotic, ultra-low oxygen conditions, whereas the functionality of the truncated protein is increasingly compromised in free-living cells under 1% and 3% O_2_. Preliminary analysis of the mutant protein indicates that it still binds HupL, although at lower level, whereas it appears as fully competent in HupK binding (data not shown).

The results presented in this work indicate the exis-tence of physical interactions between HupF, HupK, and HupL during biosynthesis of the hydrogenase large subunit in *R. leguminosarum*. This subunit contains cysteine motifs involved in the binding of the NiFe cluster [1]. The identification of similar motifs in HupK-like proteins had led to the hypothesis of a scaffolding role for HupK similar to that of NifE protein in nitrogenase synthesis [36]. Experimental evidence showed that HoxV, the HupK homolog in the *R. eutropha* system, was indeed able to bind the cofactor precursor with the cyano- and carbonyl ligands bound to a Fe atom, thus assigning a key role to this protein in the incorporation of the cofactor into hydrogenase [20]. In the same system, the existence of HoxL-HoxG and HypC-HoxV complexes was inferred from SDS-PAGE analysis of proteins obtained in co-purification experiments [20]. The data from immunoblot analysis under native conditions and from mass spectrometry analysis presented here provide a direct evidence of the existence of two such complexes in *R. leguminosarum*: a major HupL-HupF complex and a much less abundant one involving HupF and HupK. The high recovery of HupL with HupF_ST_ points towards a strong interaction between both proteins in the Δ*hupD* mutant, where the NiFe cofactor is supposed to be inserted into HupL but the protein is still unprocessed. In this situation HupF is firmly attached to unprocessed HupL, and we hypothesize that this immature protein might require the oxygen-protective function of HupF to protect the labile NiFe cluster prior to proteolytic processing, when the protein is still in an open conformation. Following the model described for the *R. eutropha* system [24] we propose that *R. leguminosarum* proteins in these complexes interact to transfer the iron-containing hydrogenase cofactor precursor from HupK to HupL, prior to the final HupD-mediated proteolytic step. But HupF protein also contributes to the stability of hydrogenase large subunit at high oxygen tensions. Data from experiments performed in a Δ*hupS* background indicate that HupF is not bound to HupL after HupD-mediated proteolytic processing (our unpublished results), indicating that mature HupL is stable enough to not require any additional chaperones, as suggested also by the results on stability of mature enzyme under 21% O_2_ presented in this paper. This model might not be the only possibility for the biosynthesis of oxygen-tolerant hydrogenases, since recent evidences indicate that hydrogenase-1 from this *E. coli* is an oxygen-tolerant hydrogen uptake hydrogenase [37], and neither HupF- nor HupK-like proteins are present in this bacterium.

Previous data from our lab and from other laboratories suggest that adaptations to the presence of oxygen also exist for the synthesis of hydrogenase small subunit HupS through the participation of HupGHIJ proteins or their homologues [19,38]. In the case of endosymbiotic bacteria, such as *R. leguminosarum*, the synthesis of hydrogenase under the ultra-low oxygen tensions prevalent in symbiotic conditions is less severely dependent on such auxiliary proteins [19]. The low, although significant, levels of hydrogenase activity detected in bacteroids induced by the Δ*hupF* mutant, but not in vegetative cells, might indicate that for *R. leguminosarum* this major HypC -> HupK -> HupF -> HupL pathway for cofactor transfer might coexist in bacteroids with a low level of alternative biosynthesis, perhaps *via* the direct HypC -> HupL mode established for *E. coli*[2].

The assembly and incorporation of non-protein ligands is a critical aspect in hydrogenase synthesis for which we still have a limited knowledge. The newly described role for HupF in this process is probably one of the adaptations to the presence of oxygen, a condition that likely affected the evolutionary history of this metalloenzyme originated in an ancient, mainly anaerobic period of the biosphere. A better understanding of the molecular basis of these adaptations will hopefully allow the design of oxygen tolerant hydrogenase enzymes for biotechnological purposes.

## Conclusions

Analysis of mutants induced for hydrogenase activity under different conditions indicate that HupF has a dual role during hydrogenase biosynthesis: it is required for hydrogenase large subunit processing, and also acts as a chaperone to stabilize HupL when hydrogenase is synthesized in the presence of oxygen. The HupF-HupL and HupF-HupK complexes identified in pull-down experiments and mass spectrometry analysis are likely involved in such functions.

## Methods

### Bacterial strains, plasmids, and growth conditions

Strains and plasmids used in this study are listed in Table 3. *R. leguminosarum* strains were routinely grown at 28°C in YMB [39]. *E. coli* DH5α was used for standard cloning procedures and *E. coli* S17.1 for conjugative plasmid transfer between *E. coli* and *R. leguminosarum*. Antibiotic concentrations used were as follows (μg ml^-1^): ampicillin, 100; kanamycin, 50; tetracycline, 5 (for *R. leguminosarum*) or 10 (for *E*. *coli*).

**Table 3:** Bacterial strains and plasmids used in this work

**Strain or plasmid**	**Relevant genotype or phenotype**	**Source or reference**
*Rhizobium leguminosarum*		
UPM791	128C53 wild type; Str^r^ Nod^+^ Fix^+^ Hup^+^	[40]
UPM1155	UPM791 ( Δ*hup*/*hyp* cluster) Hup^-^	[19]
*Escherichia coli*		
DH5_α_	*recA1 endA1 gyrA96 thi hsdR17 supE44 relA1* Δ(*lacZYA*-*argF*)*U169* Φ80d*lacZ*ΔM15	[41]
S17.1	*thi pro hsdR*^*-*^*hsdM*^*+*^*recA* RP4::2-Tc::Mu-Kan::T7; (Sp^r^ Sm^r^)	[42]
Plasmids		
pAL618	pLAFR1-based cosmid containing the whole *R. leguminosarum* hydrogenase gene cluster	[40]
pALPF1	pAL618 with *hupSL* promoter replaced by *fixN* promoter (P_*fixN*_*)*	[18]
pALPF2	pALPF1 Δ*hupL*	[19]
pALPF4	pALPF1 Δ*hupD*	[19]
pALPF5	pALPF1 Δ*hupF*	This work
pALPF10	pALPF1 Δ*hupK*	This work
pALPF14	pALPF1 Δ*hypC*	This work
pALPF382	pALPF1 derivative carrying *hupF*_*ST*_ gene	This work
pBBR1MCS-2	Broad-host-range plasmid; Km^r^ mob^+^	[43]
pKD3	Template plasmid harbouring FLP-mediated excision sequences flanking Cm^r^ gene	[44]
pPM71	PKD3 derivative containing *Strep*-tag II sequence for C-terminal end fusion	This work
pPM1350	pBBR1MCS-2 derivative containing a DNA fragment harbouring P_*fixN*_ promoter from *R. leguminosarum*	[19]
pPM501	pPM1350 derivative containing an *Nde*I-*Xba*I fragment harbouring *HupF*_*ST*_ under the control of P_fixN_	This work
pPM501C	pPM501 derivative containing a deletion of the 25 3′codons of *hupF*	This work
pPCR2.1-TOPO	PCR cloning vector	Invitrogen

### Plant tests

Pea (*Pisum sativum* L. cv. Frisson) seeds were surface-disinfected, pregerminated on agar plates, sown in Leonard jar-type assemblies, and inoculated with *R. leguminosarum* bv. *viciae* strains, as previously described [45]. Plants were grown for 21 days under bacteriologically controlled conditions with a nitrogen-free plant nutrient solution in a greenhouse adjusted to 18/25°C(night/day) temperatures. Nitrogen-free plant nutrient solution was supplemented with 170 μM NiCl_2_ on day 10 after seedling inoculation. Bacteroid suspensions were obtained from nodules as previously described [40].

### Hydrogenase activity assays

Hydrogenase activity in bacteroid suspensions and in free-living microaerobic cell cultures was measured by an amperometric method using a Clark-type electrode with oxygen as electron acceptor [45]. Hydrogenase activity in vegetative cells was induced in 40-ml cultures grown under continuous bubbling with a gas mixture containing O_2_ concentrations of 1 or 3% in N_2_. Strains were aerobically grown in YMB medium to an optical density at 600 nm (OD_600_) of *ca*. 0.4. From these cultures a 1:4 dilution was made in fresh YMB medium. Flasks were capped with a stoppered-tube system adapted to continuous flushing with 1% or 3% O_2_ on N_2_, and incubated at 28°C for 20 h. For HupL stability studies, bacteri-al cultures were maintained in a bottle with continuous bubbling with either 1% O_2_ or air during 3 hours after standard microaerobic induction (1% O_2_). Cell cultures were centrifuged and suspended in 5 ml Dixon buffer (32 mM K_2_HPO_4_, 24 mM KH_2_PO_4_ and 0.24 mM MgCl_2_) before amperometric determinations. To prevent dam-age of hydrogenase due to O_2_ exposition, extracts were bubbled with argon during preparation. Protein contents of vegetative cells and bacteroids were determined by the bicinchoninic acid method [46] after alkaline digestion of cells at 90°C in NaOH for 10 min, with bovine serum albumin as the standard.

### DNA manipulation techniques and mutant construction

DNA manipulations, including purification, restriction, ligation, agarose gel electrophoresis, PCR amplification, and transformation into *E. coli* cells were carried out by standard methods [47].

In-frame deletions of *hupF, hupK* and *hypC* genes were generated in plasmid pALPF1 as described by Manyani et al. [19], resulting in plasmids pALPF5, pALPF10, and pALPF14, respectively. Primers used for deletions and plasmid generation are included in Table 4.

**Table 4:** Oligonucleotides used in this work

**Primer**	**Sequence (5**^**′**^**-3**^**′**^**)**	**Use**
HUPF5	GCGGCACTGCTGGTCGGCTGAATCACTCCCAAGGCTGAGGTGTAGGCTGGAGCTGCTTC	*hupF* deletion
HUPF3	CGGCGGCAATTCCGGCTCGCGGGAAATGAGATCGGCGAACATATGAATATCCTCCTTAGT	
HUPK52	ACATTCCTTCTCGGGGCCGGCACGATAGGGATCGACGTGATTCCGGGGATCCGTCGACC	*hupK* deletion
HUPK32	TGCGATAGGCTGCGAGCCTGCCGTCACCGCCGATTTCGAGTGTAGGCTGGAGCTGCTTC	
HYPC5	GAACGAACATGCATCAATCGAGGAGAACCGGACATGTGCATTCCGGGGATCCGTCGACC	*hypC* deletion
HYPC3	TCGTCGATATATTTCATGCCGCATCTCCCATCGCACGTTGTGTAGGCTGGAGCTGCTTC	
TAGF31	TCCCGCGAGCCGGAATTGCCGCCGCATTTGCGCGGCGAGTGGAGCCACCCGCAGTTCGA	Generation of *hupF*_*ST*_ fusion (pALPF382)
TAGF32	GGTGGAAACTCAAATTCCATTTTGGAAGTTCTCTTTTCAATATGAATATCCTCCTTAGT	
FNDE	CTGTCAGTCGTCATATGTGCATCGGCATTCCCAT	Generation of pPM501/pPM501C plasmids
MANG3	ACGGCGGCGGGAATGCTC	
HUPF-3413 L-Strep	GAGTACTCTCAGGCGCCTTTTTCGAACTGCGGGTGGCTCCAGCTAGCTCCCTCCACCGCTTCGGCAAGTCCGGCGAG	Generation of pPM501C plasmid

To generate the HupF*::Strep*TagII (HupF_ST_) fusion protein, a modification of the Datsenko and Wanner deletion system [44] was used. The modification consisted in insertion of the sequence coding for the *Strep*Tag II peptide (WSHPQFEK) in the 5′end of the antibiotic resis-tance gene of the pKD3 plasmid [19] resulting in plasmid pPM71. This plasmid was used as template for in-frame fusing of the *Strep*Tag II sequence to the 3′ end of *hupF* from pALPF1 plasmid using TAGF31-TAGF32 by a procedure previously described [19]. The resulting pALPF1 derivative plasmid pALPF382 harbors a hydrogenase gene cluster encoding *hupF*::*Strep*Tag II (*hupF*_*ST*_*)*.

In order to express *hupF*_*ST*_ gene in microaerobically grown cultures of *R. leguminosarum* in a compatible way with Hup expression from pALPF1 derivatives, a pBBR1MCS derivative plasmid (pPM501) harboring *hupF*_*ST*_ was constructed. To this end we amplified this gene using plasmid pALPF382 as template and FNDE-MANG3 primers. Amplified fragment was cloned (*Nde*I-*Xba*I) in pPM1350 plasmid [19]. This plasmid harbors the P_*fixN*_ promoter from pALPF1 that is expressed in microaerobic conditions under the control of the FnrN protein.

A truncated form of HupF_ST_ lacking the C-terminal region (HupF_CST_) was generated by using plasmid pALPF1 as template for the in-frame deletion of the 25 codons at the 3' end of *hupF* gene. The sequence coding for the *Strep*TagII peptide was fused in frame to the corresponding site of *hupF* using primers FNDE and HUPF-3413 L-Strep. Amplified DNA was cloned in PCR 2.1-TOPO, and the construct was confirmed by sequencing. Then, the DNA region containing the truncated *hupF* gene (*hupF*_*CST*_) was excised with *Nde*I and *Xba*I and cloned downstream the P_*fixN*_ promoter of plasmid pPM1350, resulting in plasmid pPM501C. For this cloning we took advantage of the *Nde*I site generated with primer FNDE and the *Xba*I site from plasmid PCR2.1.-TOPO.

### Purification of HupF-*Strep*Tag II fusion protein

Protein purification was carried out from 3 l of bacterial cultures of *R. leguminosarum* induced for hydrogenase activity under continuous bubbling with a 1% O_2_ gas mixture. 40 ml portions of cultures were centrifuged, and cells were resuspended in 5 ml Dixon buffer and assayed for hydrogenase activity as described before. Cell suspensions and extracts used for protein purification were bubbled with argon to avoid damage of hydrogenase from O_2_ exposure, and centrifuged at 6000 rpm at 4°C for 10 minutes. The pellets were suspended in 2 ml of buffer W (100 mM Tris–HCl, pH 8, 150 mM NaCl) containing a protease inhibitor mixture (Complete-mini; Roche Diagnostics GmbH). Cells were disrupted by three passages using a French pressure cell (SLM Aminco, Silver Spring, MD) at 100 MPa and soluble fractions were cleared from cell debris and membranes by ultracentrifugation at 135,000 × g at 4°C for 1 h. The supernatant (soluble extract) was added to a 0.2-ml *Strep*Tactin Superflow column (IBA, Göttingen, Germany) operated by gravity flow. The column was washed five times with 400 μl of buffer W to remove unbound proteins, and the tagged protein was eluted by the addition of 600 μl (6 × 100 μl) of buffer W supplemented with 2.5 mM D-desthiobiotin. Relevant fractions were pooled and concentrated using a centrifugal filter device (Amicon Ultra 0.5 ml, 3 K).

### Western immunoblot and peptide mass fingerprinting

Proteins were resolved by either standard sodium dodecyl sulfate-polyacrylamide gel electrophoresis (SDS-PAGE) or native PAGE in commercial gradient 4-20% polyacrylamide gels (Bio-Rad, Hercules, California, USA), and were transferred onto Immobilon-P membrane filters (Millipore, Bedford, MA, USA) as previously described [48]. HupL, HupK and HypB proteins were detected immunologically using antisera raised against *R. leguminosarum* HupL (1:400 dilution), HupK (1:100 dilution) and HypB (1:2,000 dilution). Blots were developed by using a secondary goat anti-rabbit immunoglobulin G-alkaline phosphatase conjugate and a chromogenic substrate (bromochloroindolyl phosphate-nitro blue tetrazolium) as recommended by the manufacturer (Bio-Rad Laboratories, Inc. Hercules, CA, USA). For HupF_ST_ identification we used *Strep*Tactin conjugated to alkaline phosphatase (1:2,500; IBA, Göttingen, Germany). Immunoblot analyses were performed with 60 μg and 20 μg (total protein) of vegetative cells and bacteroids crude extracts, respectively, for HupL, or 10 μg for HypB detection. For purification of HupF_ST_ protein and study of interactions, immunoblot analysis was performed with 4 μg of protein from pooled eluate fractions and 60 μg of protein from soluble fraction samples. For identification of complexes by peptide mass fingerprinting, 20 μg (total protein) of pooled desthiobiotin-eluted fractions from bacterial cultures of *R. leguminosarum* UPM1155(pALPF4, pPM501) were resolved in native 4–20% gradient polyacrylamide gels. Then, gels were stained by Coomassie brilliant blue G-250, and bands were excised and sent to the CBGP proteomics facility for analysis by mass spectrometry on a Kratos MALDI-TOF MS apparatus (Kratos Analytical, Manchester) after trypsin digestion. Peptide profile was compared to MASCOT database supplemented with sequences from UPM791 *hup/hyp* gene products.

## Competing interests

The authors declare that they have no competing interests.

## Authors' contributions

MA carried out most of the experimental work and constructed the C-terminal deletion mutant. HM constructed most of the mutants and plasmids and performed initial analysis of protein-protein interactions. AB conceived the experiments on HupL stability. BB performed experiments with HupF mutant proteins. JI and TRA participated in the design of the study and in the final writing of the manuscript. JP coordinated the study and drafted the manuscript. All authors read and approved the manuscript.
